# Electrical Stimulation Modulates High γ Activity and Human Memory Performance

**DOI:** 10.1523/ENEURO.0369-17.2018

**Published:** 2018-02-02

**Authors:** Michal T. Kucewicz, Brent M. Berry, Vaclav Kremen, Laura R. Miller, Fatemeh Khadjevand, Youssef Ezzyat, Joel M. Stein, Paul Wanda, Michael R. Sperling, Richard Gorniak, Kathryn A. Davis, Barbara C. Jobst, Robert E. Gross, Bradley Lega, S. Matt Stead, Daniel S. Rizzuto, Michael J. Kahana, Gregory A. Worrell

**Affiliations:** 1 Department of Neurology, Mayo Clinic, Rochester, MN 55905; 2 Department of Physiology and Biomedical Engineering, Mayo Clinic, Rochester, MN 55905; 3 Department of Psychology, Hospital of the University of Pennsylvania, Philadelphia, PA 19104; 4 Department of Radiology, Hospital of the University of Pennsylvania, Philadelphia, PA 19104; 5 Czech Institute of Informatics, Robotics and Cybernetics, Czech Technical University, Prague, 166 36 Czech Republic; 6 Department of Neurology, Thomas Jefferson University Hospital, Philadelphia, PA 19107; 7 Department of Radiology, Thomas Jefferson University Hospital, Philadelphia, PA 19107; 8 Department of Neurology, Hospital of the University of Pennsylvania, Philadelphia, PA 19104; 9 Department of Neurology, Dartmouth-Hitchcock Medical Center, Lebanon, NH 03756; 10 Department of Neurosurgery, Emory University, Atlanta, GA 30307; 11 Department of Neurosurgery, UT Southwestern Medical Center, Dallas, TX 75390

**Keywords:** brain stimulation, cognitive enhancement, ECoG, γ-activity, high-frequency oscillations, intracranial EEG

## Abstract

Direct electrical stimulation of the brain has emerged as a powerful treatment for multiple neurological diseases, and as a potential technique to enhance human cognition. Despite its application in a range of brain disorders, it remains unclear how stimulation of discrete brain areas affects memory performance and the underlying electrophysiological activities. Here, we investigated the effect of direct electrical stimulation in four brain regions known to support declarative memory: hippocampus (HP), parahippocampal region (PH) neocortex, prefrontal cortex (PF), and lateral temporal cortex (TC). Intracranial EEG recordings with stimulation were collected from 22 patients during performance of verbal memory tasks. We found that high γ (62–118 Hz) activity induced by word presentation was modulated by electrical stimulation. This modulatory effect was greatest for trials with “poor” memory encoding. The high γ modulation correlated with the behavioral effect of stimulation in a given brain region: it was negative, i.e., the induced high γ activity was decreased, in the regions where stimulation decreased memory performance, and positive in the lateral TC where memory enhancement was observed. Our results suggest that the effect of electrical stimulation on high γ activity induced by word presentation may be a useful biomarker for mapping memory networks and guiding therapeutic brain stimulation.

## Visual Abstract

**Figure:**
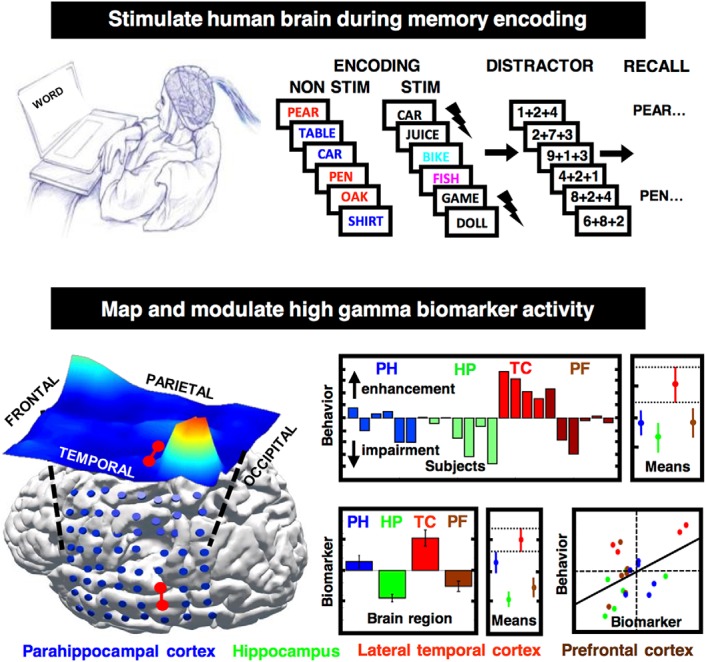

## Significance Statement

Brain stimulation technologies for memory disorders can be advanced with improved understanding of the physiologic processes modulated by electrical current. In this study, intracranial EEG recordings from epilepsy patients performing memory tasks during direct brain stimulation revealed distinct changes in the induced high γ activity, particularly on the trials with poor memory encoding. Given that these physiologic changes were correlated with the effect of stimulation on task performance, we propose they may be useful as a biomarker to optimize brain stimulation parameters for memory enhancement. These findings could help accelerate development of brain-machine interface technologies to treat memory and cognitive disorders.

## Introduction

Studies of direct electrical stimulation of the human brain were pioneered in epilepsy patients undergoing surgery to treat drug resistant focal epilepsy. During the surgery when patients were awake and stimulated in specific areas of the neocortex, they reported conscious experience of past events (Penfield, 1958). This phenomenal effect of invoking declarative memory representations was more likely to occur when stimulating in a discrete range of spectral and temporal parameters, which led to a hypothesis that the electrical current that was passed through the neural tissue activated specific neurophysiological activity supporting memory (Bickford et al., 1958; Penfield and Perot, 1963). In the current study, free recall tasks were used to investigate how stimulation in specific brain regions modulated the electrophysiological activities induced by word presentation and their subsequent recall.

Recent attempts at human memory enhancement have primarily focused on the hippocampus (HP) and the associated mesial temporal lobe structures, with reports of positive outcomes described in small studies of individual brain regions (Suthana and Fried, 2014; Kim et al., 2016). In general, however, studies have shown inconsistent results for stimulation in mesial temporal lobe structures, including: HP (Coleshill et al., 2004; Suthana et al., 2012; Fell et al., 2013; Jacobs et al., 2016), entorhinal cortex (Suthana et al., 2012; Fell et al., 2013; Jacobs et al., 2016), and fornix (Hamani et al., 2008; Miller et al., 2015). The effect of stimulation on the neurophysiological activity associated with memory tasks was largely unexplored. The positive effects of stimulation on memory reported in some of these studies were observed either in a single case (Hamani et al., 2008) or at the level of a group of patients (Suthana et al., 2012; Miller et al., 2015) without a detailed analysis of the electrophysiological signals, which is often challenging because of the stimulation artifacts (Johnson et al., 2013). In summary, limitations in the sample size, number of brain regions tested, and analysis have impeded our understanding of the impact of direct human brain stimulation on memory processes.

γ activities in the local field potential present one plausible target for exploring the neurophysiology of memory processes and the effect of stimulation. These activities have been associated with cognitive functions, including perception, attention and memory (Singer, 1993; Tallon-Baudry and Bertrand, 1999; Fries, 2009; Düzel et al., 2010). γ activities in the high frequency ranges (40–150 Hz) were proposed to be generated by local neuronal assemblies underlying cognitive processing during task performance (Crone et al., 2006; Lachaux et al., 2012), and thus provide a potential biomarker for mapping brain functions. Recent studies of γ activity in humans and nonhuman primates showed discrete bursts of γ power induced by memorized stimuli (Kucewicz et al., 2014; Lundqvist et al., 2016). In these studies, the rate of high γ burst events was associated with memory performance and proposed to underlie the differences in average power induced between trials with remembered and forgotten items, i.e., the subsequent memory effect (Kahana, 2006; Sederberg et al., 2007). Although the physiologic source of γ activities, local field oscillations or firing of neuronal assemblies, and their role in cognitive function are actively debated (Crone et al., 2006; Waldert et al., 2013; Kucewicz et al., 2017), they may still be useful as a measure of neuronal processing and modulation.

There is growing evidence that γ activities can be modulated by external interventions. Optogenetic stimulation of distinct neuron types was shown to increase γ power in the local field potential and enhance neuronal network performance in rodents (Sohal, 2016). γ power can also be increased through neurofeedback training in specific brain regions, as reported in nonhuman primate recordings that showed synchronous neuronal firing and enhanced behavioral performance (Engelhard et al., 2013). Transcranial current stimulation is another approach used to modulate γ activities and, for instance, was shown to induce dream self-awareness in the human subjects (Voss et al., 2014). The effect of direct stimulation of the human brain on γ activities linked to memory performance has been largely unexplored. However, the reports of a positive effect on memory performance in humans were all stimulating at frequencies in the γ range (40/50/200 Hz; for review, see Kim et al., 2016), suggesting that the applied current presumably modulated similar frequencies of neuronal oscillations. Here, we tested the effect of 50-Hz electrical stimulation on γ activity and task performance in four brain regions supporting declarative memory.

## Materials and Methods

### Study participants

Patients undergoing intracranial electroencephalographic monitoring as part of their clinical treatment for drug-resistant epilepsy were recruited to participate in this multi-center collaborative study. Data were collected from the following clinical centers: (Mayo Clinic, Thomas Jefferson University Hospital, Hospital of the University of Pennsylvania, Dartmouth-Hitchcock Medical Center, Emory University Hospital, University of Texas Southwestern Medical Center). The research protocol was approved by the respective IRB at each clinical center and informed consent was obtained from each participant. Electrophysiological data were collected from standard clinical subdural and penetrating depth electrodes (AdTech Inc., PMT Inc.) implanted on the cortical surface and into the brain parenchyma, respectively. The subdural electrode contacts were arranged either in a grid or a strip configuration with contacts separated by 10mm. The depth electrode contacts were separated by 1.5–10 mm spacing. In each case, the placement of the electrodes was determined by a clinical team whose sole purpose was to localize seizures for possible epilepsy surgery. In this study, we identified 22 patients (nine males) with subdural or depth electrodes implanted in at least one of the four brain regions of the cortical-hippocampal declarative memory system (Eichenbaum, 2000), who completed at least two stimulation sessions in any of these regions (Tables 1, 2).

**Table 1.** Clinical profile of the study participants

Subjectno.	Age	Gender	Handedness	SOZ	MRI	Brain pathology	Language laterality(method)	Stimulation mapping overlap	vIQ	Verbalmemorydeficits
1001	48	F	R	Right TC	Normal	Gliosis	L (fMRI)	-	81	None
1006	20	F	R	Right FC	MCD	Gliosis	L (fMRI)	-	91	None
1016	31	F	R	Left FC	Normal	Gliosis	-	None	71	None
1018	47	M	L	Left FC,left FPC	Normal	-	L (fMRI)	-	85	None
1020	48	F	L	Right TC,right FC	Abnormal	Gliosis	L (fMRI)	-	98	Mild
1022	24	M	R		AtrophyGliosis/encephalomalacia	-	L (fMRI)	-	81	None
1024	36	F	R	Right OPC	Normal	Gliosis	L (unknown)	-	100	None
1026	24	F	R	Left aTCleft OC	MTS, gliosis	-	Bilateral (Wada)	-	112	None
1027	48	M	R	Right TC right ICright/left FC	Abnormal	-	L (fMRI)	-	93	None
1028	27	F	R	Right MTL	Abnormal	CD, Gliosis	L (Wada)	-	103	None
1029	33	F	R	Left FC	Abnormal	-	-	-	108	Mild
1030	23	M	L	Left MTL	Normal	Gliosis	L (fMRI)	-	106	None
1031	24	M	R	Right FC right TC	Abnormal	-	L (aphasia)	-	110	Moderate
1033	31	F	R	Right TC	Atrophy	-	L (Wada)	-	85	None
1036	49	M	L	Left aTC,left MTL	MTS	HS	Bilateral (Wada)	-	93	Moderate
1042	27	F	L	Right TC	MCD	-	R (fMRI)	None	114	None
1050	20	M	R	Left PC	Neoplasm	DNET	Bilateral (Wada)	None	95	Mild
1060	36	F	R	Right TC	Normal	Gliosis	L (Wada)	-	95	Mild
1069	26	M	R	Left FC	MCD	-	L (Wada)	-	-	Mild
1111	20	M	R	Left TC left OPCleft OC	Gliosis	Gliosis	L (fMRI)	-	108	None
1176	41	F	R	Right MTL right IC	MTS	-	L (Wada)	-	85	Moderate
1177	23	F	R	Left TC	TS	-	L (aphasia)	None	87	Moderate

Patient demographic data are presented together with clinical observations from structural MRI, clinically identified seizure onset zones (SOZs), pathology for those subjects who underwent respective surgery, hemispheric laterality of language functions together with the method of determination (“aphasia” means that the determination was done based on an identified lesion/pathology in a specific hemisphere), overlap of the stimulating electrodes with the language areas for patients who have undergone cortical stimulation mapping (“-”; means that the stimulation mapping was not performed or the report was not available), verbal IQ (vIQ), and the clinical qualitative description of verbal memory deficits as concluded in the neuropsychological assessment. FC, frontal cortex; PC, parietal cortex; OC, occipital cortex; IC, insular cortex; aTC, anterior TC; MTL, mesial temporal lobe; TPC, temporo-parietal cortex; FPC, fronto-parietal cortex; OPC, occipito-parietal cortex; CD, cortical dysplasia; HS, hippocampal sclerosis; MCD, malformation of cortical development; MTS, mesial temporal sclerosis; PMG, polymicrogyria; DNET, dysembryoplastic neuroepithelial tumor.

**Table 2.** Summary of the experiments used to assess effect of stimulation on encoding of word lists

Subject	Sessions	Localization	Region	Electrode	Amplitude
1001	2	Left HP	HP	Depth	1.0
1006	2	Right HP	HP	Depth	1.0
1016	2	Left PF	PF	Subdural	3.5
1018	2	Left PF	PF	Depth	1.5
1020	4	Right HP	HP	Depth	1.0
1022	2	Left HP	HP	Depth	1.0
1024	3	Left HP	HP	Depth	1.0
1026	4	Left EC	PH	Depth	0.5
1027	2	Left HP	HP	Depth	1.0
1028	3	Right EC	PH	Subdural	1.0
1029	2	Left PF	PF	Subdural	3.5
1030	4	Left PHC	PH	Depth	0.5
1031	2	Right PRC	PH	Depth	1.5
1033	2	Left PRC	PH	Depth	1.5
1036	4	Left PRC	PH	Depth	1.0
1042	2	Right PF	PF	Subdural	1.5
1050	2	Left TC	TC	Subdural	1.5
1060	3	Right PF	PF	Subdural	3.0
1069	2	Left PF	PF	Subdural	2.5
1111	3	Left PHC	PH	Depth	0.75
1111	3	Left TC	TC	Subdural	1.5
1176	3	Left TC	TC	Depth	1.0
1177	4	Left TC	TC	Subdural	1.0

Analysis was focused on 23 subject experiments that had at least two sessions with any one stimulation target in four of the studied brain regions. PHC, PH cortex; PRC, perirhinal cortex; EC, entorhinal cortex.

### Anatomic localization and brain surface mapping

Cortical surface parcellations were generated for each participant from preimplant magnetic resonance imaging (MRI) scans (volumetric T1-weighted sequences) using Freesurfer software (RRID:SCR_001847; Fischl et al., 2004). The HP and surrounding cortical regions were delineated separately based on an additional 2-mm-thick coronal T2-weighted scan using the Automatic Segmentation of Hippocampal Subfields (ASHS) multi-atlas segmentation method (Yushkevich et al., 2015). Electrode contact coordinates derived from registered postimplant CT scans were then mapped to the preimplant MRI scans to determine their anatomic locations. For subdural strips and grids the electrode contacts were additionally projected to the cortical surface using an energy minimization algorithm to account for postoperative brain shift (Dykstra et al., 2012). For comparisons across subjects, coordinates were transformed to the MNI brain space, in which distance between bipolar electrode pairs was estimated using the shortest path from the stimulating electrode pair. Contact locations were reviewed and confirmed on surfaces and cross-sectional images by a neuroradiologist. For further visualization and presentation purposes, surfaces and contact coordinates were rendered using Blender (http://blender.org) and Blend4web (http://blend4web.org) open source software in a customized interactive web application.

### Electrophysiological recordings

Intracranial data were recorded using one of the following clinical electrophysiological acquisition systems specific to a given site of data collection: Nihon Kohden EEG-1200, Natus XLTek EMU 128, or Grass Aura-LTM64. Depending on the acquisition system and the preference of the clinical team, the signals were sampled at either 500, 1000, or 1600 Hz and were referenced to a common contact placed either intracranially, on the scalp, or on the mastoid process. For analysis all recordings using higher sampling rates were down-sampled to 500 Hz. A bipolar montage was calculated *post hoc* for each subject by subtracting measured voltage time series on all pairs of spatially adjacent contacts. This resulted in *N* – 1 bipolar signals in case of the penetrating and the strip electrodes, and *N* + x bipolar signals for the grid electrodes, where *N* is the number of electrode contacts and x is the number of extra combinations of bipolar contacts that resulted from the montage.

### Memory tasks with brain stimulation

**Figure 1.**
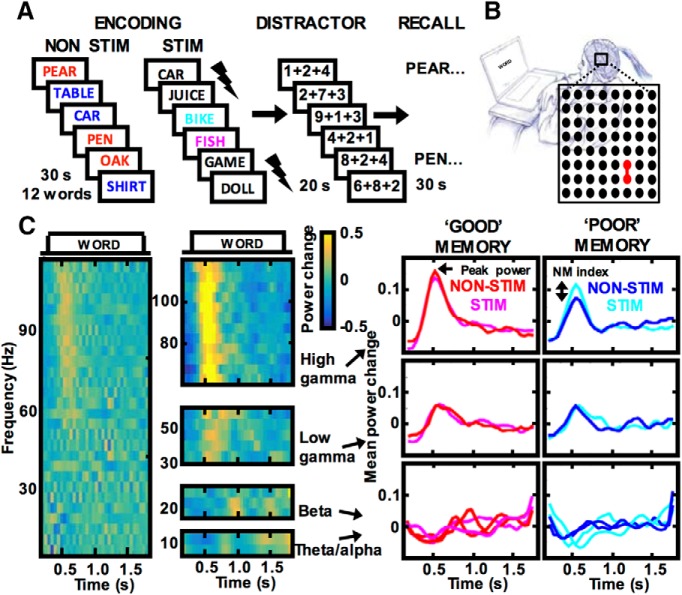
Free recall tasks to study electrophysiological modulation of verbal memory encoding. ***A***, Diagram of the task design, in which subjects memorized word lists for subsequent recall. Thunderbolt marks the words with stimulation on the STIM lists. The remaining word trials were used for electrophysiological analysis and are labeled according to the lists type (NON-STIM or STIM) and their encoding based on subsequent recall (GOOD or POOR). ***B***, Example of an 8 × 8 electrode grid implanted over the lateral TC highlights in red two adjacent contacts used for brain stimulation (connected red dots) in subject 1050. ***C***, Broadband spectrogram (left column) shows trial-averaged power changes aligned to the time of word presentation for encoding, in contrast to the power changes in the signal prefiltered in the four studied frequency bands (middle column), as recorded from a representative electrode example from subject 1111. Line plots on the right summarize the mean power change response independently for the four bands (rows) and separately for the good and poor encoding trials (columns) in the two conditions of list stimulation, color-coded as in ***A***. Notice the difference in peaks of the response (NM index) caused by stimulation in the poor encoding trials specifically in the high γ frequency band.

The tasks were based on classic paradigms for probing verbal memory (Kahana, 2012), in which subjects learned lists of words for subsequent recall (Fig. 1*A*). Subjects were instructed to study lists of individual words presented sequentially on a laptop computer screen for a later memory test. Lists were composed of 12 words chosen at random and without replacement from a pool of high frequency nouns (either English or Spanish, depending on the participant's native language; http://memory.psych.upenn.edu/WordPools). Each session had a set of 25 specific lists using words from the same general pool. The words on each list were either sampled from specific categories like vehicles, music instruments and vegetables, or they were sampled randomly. Each word remained on the screen for 1600 ms, followed by a random jitter of 750- to 1000-ms blank interval between stimuli. Immediately following the final word in each list, participants performed a distractor task (20 s) consisting of a series of arithmetic problems of the form “A + B + C = ??”, where A, B, and C were randomly chosen integers ranging from 1 to 9. Following the distractor task subjects were given 30 s to verbally recall as many words as possible from the list in any order. Vocal responses were digitally recorded by the laptop computer and later manually scored for analysis. Each session consisted of 25 lists of this encoding-distractor-recall procedure.

Stimulation was applied by passing electrical current between two adjacent electrode contacts using parameters from the study (Suthana et al. 2012) showing a positive effect of stimulation on memory performance (bipolar symmetric, charge-balanced, square-wave stimulation at a frequency of 50 Hz and 300-μs pulse width). Safe amplitude for stimulation was determined at the start of each session using a mapping procedure in which stimulation was applied at 0.5 mA while a neurologist monitored for after-discharges. This procedure was repeated, incrementing the amplitude in steps of 0.5 mA, up to a maximum of 1.5 mA for depth contacts and 3.5 mA for cortical surface contacts. These maximum amplitudes were chosen to be below the after-discharge threshold and below accepted safety limits for charge density (McCreery et al., 1990). The stimulation was delivered for 4600 ms during the presentation of two subsequent words (from 200 ms before the first word onset to 200–450 ms after second word offset due to a random jitter in inter-stimulus interval) on every other word pair (three pairs on every list with first pair pseudorandomized across all lists in a given session). Stimulation was applied on 20 out of 25 randomly assigned lists of a full session. There were no more than two sessions a day of a given task separated by at least three hours. The target electrode pair for stimulation was selected based on the anatomic coverage of brain regions associated with declarative memory functions (Eichenbaum, 2000), including hippocampus (HP), parahippocampal region (PH), temporal cortex (TC), and prefrontal cortex (PF). Within these regions specific target electrode pairs for stimulation were selected based on anatomic localization in one the studied brain regions and based on mapping of active areas showing a subsequent memory effect (Kahana, 2006; Sederberg et al., 2007). Electrodes had to be localized outside the seizure onset zone, as defined by the local clinical team. Additional clinical data were collected about the localization of language functions relative to the stimulation sites and neuropsychological assessment of verbal memory (Table 1). Stimulation amplitude was determined using conservative limits for safe charge density (Gordon et al., 1990; McCreery et al., 1990) for subdural or depth electrode contact, not higher than 3.0 and 1.5 mA, respectively.

### Electrophysiological analysis

Brain activity induced by word presentation was analyzed in this study, and comprised 1600 ms of word display on the screen and 200-ms blank interval before and after each word (total 2000 ms segments). Stimulated word pair epochs were excluded from analysis to prevent potential contamination of spectral analysis with the stimulus artifact. Hence, one complete session yielded electrophysiological signal from 60 nonstimulated list epochs (five lists × 12 words) and 120 stimulated list epochs (20 lists × six words). Every signal epoch was spectrally decomposed in 50-ms time bins using multi-taper Fast Fourier Transform (Chronux toolbox, RRID:SCR_005547; Bokil et al., 2010); taper parameters: 4-Hz bandwidth, 250-ms timewidth, one taper). To estimate power in distinct frequency bands (high γ: 62–118 Hz, low γ: 30–58 Hz, β: 14–26 Hz, θ/α: 6–14 Hz) signals were bandpass filtered between the corresponding cutoff frequencies (Barlett-Hanning, 1000 order) before spectral decomposition to reduce any possible influence of lower frequencies on the power estimate. The cutoff frequencies for the high γ band were chosen to minimize contamination of the 60-Hz line noise and its first harmonic at 120 Hz. The decomposed spectral power values in a given frequency band were log and *z*-score transformed in each frequency bin to account for the power law effect and obtain values that can be compared in the same scale across sessions and subjects. Frequency bands in the low θ and δ ranges between 1 and 5 Hz were not included in this study due to different high-pass filters applied in signal acquisition across the data collection centers. Average power estimates were calculated from all epochs of the studied words from nonstimulated lists.

Exact time of memory encoding during the stimulus presentation is difficult to determine and can vary between subjects. We used the maximum peak value of the average power estimates as proxy for the brain response related to the memory encoding. This maximum value of the average power estimate was defined as peak power, and the difference between peak power values from the stimulated (P_stim_) and nonstimulated (P_non_) list condition was defined as the “;neuromodulation (NM) index”:

NM=Pstim −Pnon

Pstim=f(t);0<t<2000ms

Pnon=f(t);0<t<2000ms

$$f(t)=a_{0}+\frac{\overset{N}{\underset{n=0}{\sum }}f(a_{n})}{N},\;where\;f(a_{n})\;is\;the\;nth\;power\;estimate.$$

Surface plots were created using the peak power and the NM index values interpolated between all bipolar pairs on an electrode grid. Active electrodes were selected by identifying outliers of the peak power value distributions above the upper adjacency value (UAV; > third quartile + 1.5 × interquartile range), which were calculated from all nonstimulated list epochs for every electrode in a given patient. The identified active electrodes were used to determine mean value of the NM index across all electrodes in a given subject or brain region, which had active electrodes from at least two subjects.

### Behavioral analysis

Memory performance was quantified as count of words recalled per list (with or without stimulation). To compare the effect of stimulation on performance across subjects the raw counts from all sessions in a given subject were normalized into *z*-scores. Difference between means of the scores on the stimulated and nonstimulated lists was defined as a measure of stimulation's effect on memory performance (Δ behavioral score). At least two sessions in a given stimulation target were required to be included in data analysis to ensure an accurate estimate of the mean for the nonstimulated lists, i.e., more than five scores were required to estimate the mean.

### Statistical analysis

All statistical tests were performed in Matlab (MathWorks Inc., RRID:SCR_001622) using built-in and custom written codes. One-way ANOVA test compared NM index calculated from the same set of electrodes from one subject in different frequency bands (Fig. 3*C*). The test was followed by Tukey–Kramer *post hoc* group comparison of the 95% confidence intervals of the means. Pearson's correlation was chosen to test dependence between NM index and: peak power value (Fig. 3*D*), distance from the stimulating electrode (Fig. 3*D*), and the behavioral effect of stimulation on memory performance (Fig. 5*D*). For the former two the correlation was additionally confirmed on the level of electrodes from individual patients. The correlation plots were complemented with least-squares lines to aid visual interpretation. ANOVA test was used to compare the effect of stimulating in the four studied regions on the NM index and on behavioral performance. The test was followed by Tukey–Kramer *post hoc* group comparison of the 95% confidence intervals of the means. Data are shown as mean ± SEM. ANOVA tables are summarized in Table 3. All data collected in this project are available at: http://memory.psych.upenn.edu/RAM_Public_Data.

**Table 3.** Statistical tables for the analyses of variance

Source of variation	Sum od squares	Degrees of freedom	Mean squares	*F* ratio	Probability > *F*
NM index in different frequency bands during the poor encoding trials					
Groups	0.10172	3	0.03391	14.79	<0.0001
Error	0.19262	84	0.00229		
Total	0.29434	87			
NM index in different frequency bands during the good encoding trials					
Groups	0.01226	3	0.00409	1.71	0.1708
Error	0.20053	84	0.00239		
Total	0.21279	87			
Effect of stimulation in different brain regions on memory performance					
Groups	1.83311	3	0.61104	7.31	0.0019
Error	1.58778	19	0.08357		
Total	3.4209	22			
Effect of stimulation in different brain regions on NM					
Groups	0.26765	3	0.08922	23.27	<0.001
Error	0.74363	194	0.00383		
Total	1.01128	197			

## Results

We investigated the effect of direct brain stimulation on electrophysiological activity and memory performance in epilepsy patients undergoing evaluation for surgery to treat refractory seizures. Each patient was implanted with intracranial subdural, depth, or subdural and depth electrode arrays in multiple cortical and subcortical brain regions selected based solely on the clinical considerations. We identified 22 patients who were implanted in one of the four brain regions of the declarative memory system (Eichenbaum, 2000) and completed at least two sessions of free recall tasks with stimulation (Tables 1, 2). The tasks were based on a classic paradigm for probing verbal short-term memory (Kahana, 2012), in which subjects learned lists of twelve words to be freely recalled in any order following a distractor (Fig. 1*A*). Electrical stimulation was applied between a pair of adjacent electrode contacts during encoding of words for subsequent recall (Fig. 1*B*). Low amplitude stimulation (<1.5 mA, 50-Hz frequency, pulse width 300 µs; Table 2) was applied for 4.6 s during presentation of two consecutive words, followed by presentation of two other words without any stimulation to enable electrophysiological analysis without stimulus artifact (Fig. 1*A*).

We found that stimulation in the lateral TC modulated the spectral power specifically in the high γ band (62–118 Hz) on electrodes showing induced responses to word presentation (Fig. 1*C*), which was associated with enhanced memory performance (for behavioral analysis, see Fig. 5). The high γ response on trials with words that are subsequently not recalled (“;poor” encoding) is known to be decreased relative to trials with the subsequently recalled words (“;good” encoding), as previously described (Kahana 2006, Sederberg et al., 2007). Stimulation on the poor encoding trials increased this high γ response and restored it to the magnitude observed on the good encoding trials with words that were subsequently recalled (Fig. 1*C*). Thus, the subsequently forgotten words from the stimulated “;STIM” lists had increased high γ response relative to the words from the “;NON-STIM” lists that were not stimulated. Each experimental session comprised of both the STIM and the NON-STIM lists, which were randomly assigned in a double-blind fashion. The modulatory effect of stimulation was quantified as a difference between peaks of the power response in the STIM minus the NON-STIM condition, which we called the NM index (Fig. 1*C*). The peak response was thus used as proxy for brain activity related to memory encoding.

**Figure 2.**
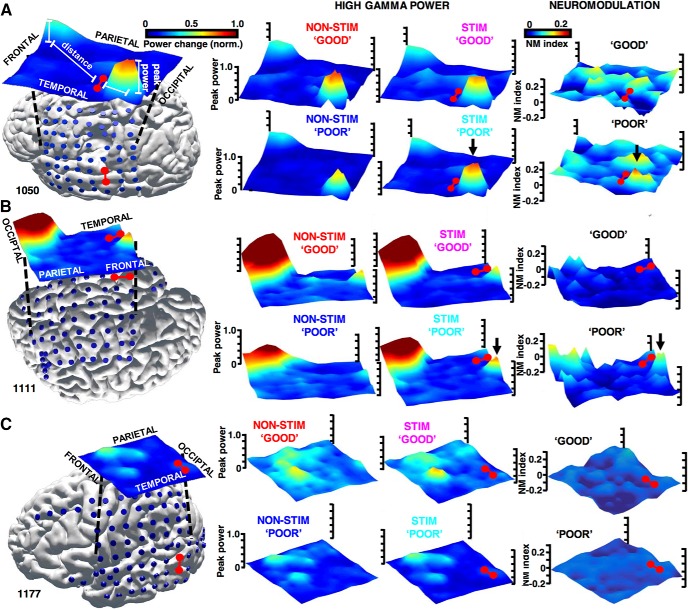
Stimulation modulates high γ responses in localized areas activated in the tasks. ***A***, Values of the peak power of the γ responses and the NM index from all 8 × 8 grid electrodes (blue dots, stimulating electrodes in red) of subject 1050, as in Figure 1*B*, are interpolated and visualized as surface plots overlaid on this subject's brain surface (left side). The first two columns present peaks of the high γ power in the STIM (first) and the NON-STIM (second) conditions, the third column presents the NM index, i.e., the effective difference between the first two columns. Arrows point to a discrete area of peak power modulated by stimulation particularly in the poor encoding trials. ***B***, ***C***, Analogous plots from two other cases of subject 1111 (brain surface rendering was turned upside down to aid visualization) and 1177, respectively. Notice that the high γ modulation is observed also at a distance from the stimulation site in subject 1111 and is not observed in subject 1177.

This NM effect was localized to “;activated” areas of the brain showing the induced high γ response in the tasks. Figure 2 presents three exemplar cases of stimulation from subdural surface grid electrodes in the TC, which modulated the peak power responses. The top case depicts a single discrete area of the peak activation. The magnitude of this discrete high γ response is greater on the good than the poor encoding trials in the NON-STIM control condition. This disparity between the remembered and the forgotten word trials is not present in the STIM condition with similar peaks on the two trial types (Fig. 2*A*). Stimulation therefore increased the high γ response on the poor encoding trials to the levels seen during good encoding, selectively in the area of the induced task activity. The middle case reveals that this effect was also observed in an activated area of the occipital cortex, which was distant from the site of stimulation located in the TC (Fig. 2*B*). We did not observe this neuromodulatory effect (quantified as the NM index) in the bottom case, where no area in the TC was activated in the tasks (Fig. 2*C*). Cortical stimulation mapping of language functions was performed as part of the clinical evaluation in patients 1050 and 1177, which showed no overlap with the target stimulation electrodes (Table 1).

**Figure 3.**
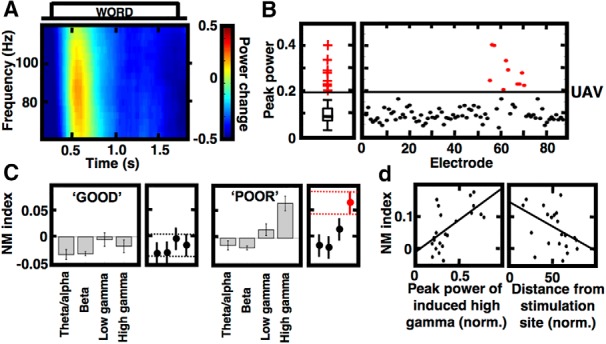
Stimulation selectively modulates task responses in the high γ frequency band. ***A***, Spectrogram of trial-averaged high γ response to word presentations recorded on an electrode in the brain area activated in the tasks. ***B***, Active electrodes showing this response were identified as positive outliers of the peak value distribution of this response (red data points above the solid line of UAV). ***C***, Mean NM index of all active electrodes in one stimulated patient (*n* = 36) is compared among four frequency bands in the poor and good memory encoding conditions. Subplots on the right show *post hoc* comparison of the group means, dashed lines mark the 95% CI intervals (error bars) for the high γ group, and red indicates significant group with the intervals that do not overlap with any other group. ***D***, Scatterplots with least-square lines show correlations of the NM index values in the poor encoding condition plotted against peak value of the task response (left) and against the distance from the stimulation site (right) for the active electrodes from ***C***. Notice that NM index was proportional to the induced power response and inversely proportional to the distance from the stimulation site.

We quantified these observations for all electrodes in the activated brain areas (“;active” electrodes) in the only stimulated subject who had more than ten such active electrodes (*n* = 22). The active electrodes were selected based on the distribution of the peak values of the high γ response from all available electrodes in a given patient (Fig. 3*A*,*B*). To test whether the observed modulation was specific to the high γ band we compared the NM index values in four nonoverlapping frequency bands (θ/α, β, low γ and high γ). A significant difference was found between the studied bands in the condition of poor memory encoding (*p* < 0.0001, ANOVA, *F* = 14.8, degrees of freedom = 3, 84) but not in the good memory encoding (*p* = 0.171, ANOVA, *F* = 1.71, df = 3, 84) in this subject. NM index values for the high γ band in the poor encoding condition were significantly more positive (Tukey–Kramer *post hoc* test, *p* < 0.05) than for any of the other bands (Fig. 3*C*). We further investigated whether these significantly more positive values of NM index were correlated with the amplitude of the high γ response and with the distance from the source of stimulation (Fig. 3*D*). The mean NM index was positively correlated with the mean amplitude of the high γ response (Pearson's correlation, *R* = 0.627, *p* = 0.0018) and negatively correlated with the distance from stimulation site (Pearson's correlation, *R* = -0.429, *p* = 0.0461). These correlations suggest that the strength of modulation was dependent on the electrode activity in the tasks and its proximity to the site of stimulation.

**Figure 4.**
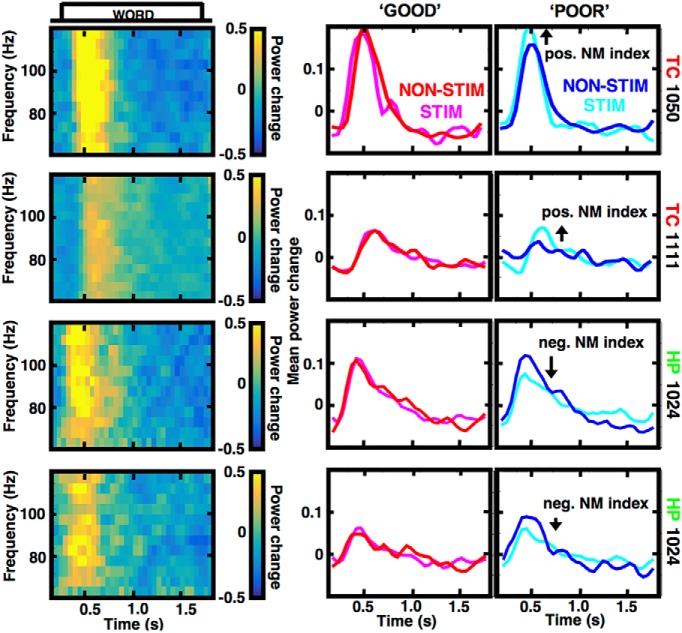
High γ responses are positively and negatively modulated in different brain regions. Four electrode examples show modulation of the task-induced high γ activities by stimulation in the lateral TC (red) and the HP (green), as presented in another example from Figure 1*C*. Arrows mark the positive and negative NM index changes in the three patients who showed the greatest positive (upper rows) and negative (lower rows) behavioral effects of stimulation (Fig. 5).

In the final part of this study, we asked whether this positive modulation of the high γ activities induced in the free recall memory tasks is specific to stimulation in the lateral TC. We observed an inverse pattern of modulation when the other studied brain regions were stimulated. Figure 4 shows two example electrodes showing a positive NM index with TC stimulation (top rows) and two negative index values with stimulation in the HP (bottom rows). The latter came from subject 1024, who noted decreased memory performance on the STIM relative to the NON-STIM lists.

**Figure 5.**
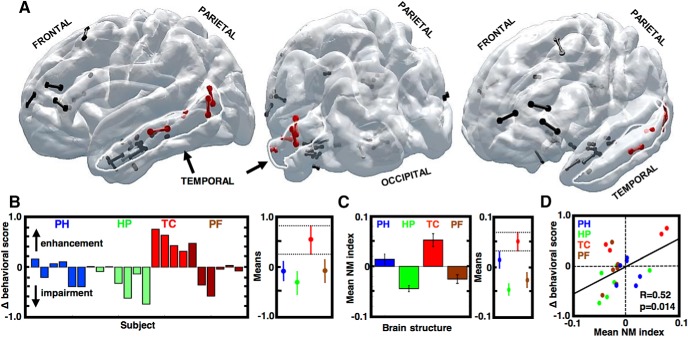
Modulation of high γ activity in different brain regions is correlated with behavior. ***A***, Localization of the stimulation sites in the lateral TC (red contact pairs) and the other three brain regions studied (black contact pairs) is visualized in a unified transparent brain surface. ***B***, Stimulation-induced change in memory performance for every subject (each bar is one subject) reveals that stimulation in the TC had a positive effect on performance compared to the other brain regions (PF). *Post hoc* group comparison (right side) shows that TC scores are greater than PH, HP, PF (dashed lines are 95% CI of the TC group). ***C***, NM index values reveal the same pattern as in ***B***, averaged from all active electrodes in a given group [*n* = 38 (PH), *n* = 80 (HP), *n* = 36 (TC), *n* = 44 (PFC)]. ***D***, The behavioral and NM index scores averaged for each subject (color-coded dots) are correlated. Least-square line is added in black, crossing the two dashed lines at point 0 indicating no stimulation-induced changes.

To test this observed relationship between the behavioral performance and magnitude of the modulation in different brain regions, we compared the effect of stimulation in the four regions involved in the declarative memory system: PH (entorhinal/perirhinal and PH gyrus), HP (subiculum and HP proper), lateral TC (middle and superior temporal gyrus), and PF (middle and inferior frontal gyrus). Precise localization of all stimulation targets used in every subject (*N* = 23) is shown on a unified brain surface (Fig. 5*A*) and can be viewed online (to be identified if the article is published). We summarized the behavioral effect of stimulation across the studied brain regions to find that all four subjects stimulated in the lateral TC showed a positive effect on memory performance (Fig. 5*B*). There was a significant effect of the brain region (*p* = 0.0019, ANOVA test, *F* = 7.31, df = 3, 19) revealing a stronger positive modulation of memory performance in the TC stimulation group than any other brain region (Tukey–Kramer test, *p* < 0.05). Stimulating in the four regions also exerted different effects on the high γ modulation (*p* < 0.001, ANOVA test, *F* = 23.27, df = 3, 194). We found that the NM index, averaged over active electrodes from stimulation in a given region (*n* = 198), followed the same pattern (Fig. 5*C*) with a stronger positive NM in the TC group compared to any other group (Tukey–Kramer test, *p* < 0.05). Plotting the behavioral modulation score as a function of the mean NM index for every subject (Fig. 5*D*) confirmed that the electrophysiological effect of stimulation and memory performance were correlated (Pearson's correlation, *R* = 0.50, *p* = 0.016). Subjects 1050 and 1111, who noted the highest NM index values, demonstrated the greatest memory enhancement (Fig. 5*D*). Conversely, subject 1024 with the lowest mean NM index, noted the greatest memory impairment.

## Discussion

In this work, we found evidence that electrical stimulation in specific regions of the human brain modulates high γ activities induced during encoding of words for subsequent recall. Positive high γ modulation, as observed with stimulation in the lateral TC, was associated with the brain region showing enhanced memory performance with stimulation, whereas negative modulation was seen in the HP, a region where stimulation had the opposite effect on memory recall. Both structures have been proposed to play differential roles in the declarative memory. HP and the medial temporal lobe structures are thought to be critical for binding episodic memory representations from distributed regions in the neocortex, which process and store memory (Squire and Zola-Morgan, 1991; Eichenbaum, 2000). Previous studies using electrical stimulation in the medial temporal lobe during memory performance in human subjects showed mixed results (Kim et al., 2016). Our results corroborate a recent report of stimulation-induced impairment in a range of tasks, including the free recall of word lists, applied in a large number of patients stimulated in the HP and the entorhinal cortex (Jacobs et al., 2016). Much less is known about the effect of stimulation in the lateral TC. Since the original reports of eliciting memory experience in individual epilepsy patients (Penfield and Perot, 1963), stimulation in this region of the human brain has been predominantly used for mapping language functions (Ojemann, 1991). Noninvasive stimulation (Tune and Asaridou, 2016) and imaging studies (Binder et al., 2009) support the role of brain regions in the lateral TC in processing semantic information. Another study with large number of epilepsy patients implanted with electrodes in various regions of the brain found that epileptiform discharges were impairing memory encoding of word lists specifically if they occurred in the lateral TC (Horak et al., 2017). Our results show that stimulation applied in the lateral TC enhanced the high γ activities in response to word encoding. In summary, there is a growing body of literature implicating the lateral TC in verbal memory functions.

Stimulation-related enhancement of the induced high γ activities was observed on trials with poor memory encoding and not on the good encoding trials. In fact, the average NM index for the high γ band during good encoding trials turned out to be negative (Fig. 3*C*). In a recent study of electrical stimulation applied during word encoding, the induced high γ activity was used to classify brain states into good and poor encoding states and predict that stimulating in the good state decreased the probability of recall and vice versa increased probability of recall when stimulating in the poor encoding state (Ezzyat et al., 2017). This interesting finding of good and poor encoding state-dependency is consistent with our observation of a positive stimulation-induced NM index during the poor encoding trials and a negative index during the good trials. Still, the positive effect of stimulation on the high γ activity was restricted to trials with words that were ultimately forgotten, making it challenging to explain the overall enhancement observed in the increased number of recalled words.

The outcome of stimulation was not only determined by the encoding brain state, but also by anatomic location. Our results show that both the neurophysiology and the behavior (recall performance) were differentially modulated depending on the brain region tested. The same stimulation pattern applied in the lateral TC versus the HP had opposite effects on the high γ responses and the associated recall performance (Fig. 5). The exact factors causing these differential effects on the neurophysiology and behavior remain unclear. The difference could be related to the qualitative differences in the electrode contacts used for stimulation, i.e., penetrating depth electrodes in the HP and subdural electrodes on lateral TC, but the surface area of the different electrodes is similar. In addition, five out of six subjects undergoing stimulation in the PF group were stimulated using subdural electrodes and did not show the same neurophysiological or behavioral effect as in the TC group. Further, the difference could be attributed to the range of stimulation parameters used. The original studies with epilepsy patients found that only a given set of amplitude and frequency parameters elicited the memory experience (Bickford et al., 1958; Penfield and Perot, 1963). Stimulating the same regions of the brain with higher amplitudes is known to disrupt cognitive processing of, e.g., verbal information mapped in these patients (Ojemann, 1991) as applied in clinical language mapping. Therefore, our reported results may not necessarily generalize to other tasks or be replicable with different set of parameters, which could not be tested within the scope of this study. Nevertheless, the results hold promise for using high γ activities as a biomarker of NM to target optimal parameters, phases and sites for stimulation and support that the stimulated region in the posterior half of the middle and superior temporal gyrus is specifically important for modulating memory processes engaged in these tasks.

Regarding the possible target sites, within the lateral TC there were distinct focal areas where word encoding induced the high γ activity (Fig. 2). These “;islands” of high frequency power have been reported in the intracranial recordings during tasks (Kucewicz et al., 2014, 2017), which may indicate local processing of neuronal assemblies (Crone et al., 2006; Lachaux et al., 2012) and be used to map target sites for stimulation. Interestingly, the precise localization of the foci of high γ activities was not exactly the same in the studied subjects even within the lateral TC, possibly due to different strategies employed by subjects in these tasks (e.g., remembering more semantic or visual representations). At this point we can only speculate about the effects of stimulating in the focus or perimeter of these islands, over a gyrus or a sulcus, or at various scales of neuronal organization. Successful stimulation sites were localized on the middle temporal gyrus adjacent to the high γ island in two out of four subjects, who showed the strongest positive effect on neurophysiology and behavior (Fig. 5). Our study as well as others in the field (Suthana and Fried, 2014; Kim et al., 2016; Jacobs et al., 2016; Ezzyat et al., 2017) were performed with standard clinical electrodes with contacts of diameters ranging from 1 to 10mm^2^ and separated by 5-10 mm. We speculate that future studies using combined macro- and micro-electrode arrays could provide additional information of the spatial scale of the neuronal networks underlying memory function (Le Van Quyen et al., 2010; Viventi et al., 2011; Worrell et al., 2012; Kucewicz et al., 2016).

With regard to the target phases and parameters for stimulation, there are many other possible approaches to enhance memory processing and task performance. We have focused on modulating the encoding of memorized stimuli during their presentation, which induces high frequency activities. Another approach is to modulate maintenance, consolidation or retrieval of memory for the encoded items, which are thought to engage oscillatory activities in the lower frequency bands, including the θ rhythm (Buzsaki, 2006; Düzel et al., 2010). These lower frequency oscillations were shown to be more widely spread than the focal γ responses (Burke et al., 2013; Kucewicz et al., 2014), thus possibly providing a viable target for noninvasive stimulation techniques. For instance, transcranial magnetic stimulation was employed to modulate θ oscillations mapped in parietal cortex to enhance retention of nonverbal memory (Albouy et al., 2017). Memory performance was increased in 13 out of 17 subjects and attributed to entrainment of the θ oscillations during the maintenance phase of the task. Other studies using noninvasive stimulation in similar tasks to probe active maintenance of memory in the PF showed mixed effects on reaction time and accuracy (Brunoni and Vanderhasselt, 2014). Although these studies are limited in terms of elucidating the ongoing neurophysiological activity, they complement the invasive intracranial recordings with insight into other measures of neural excitability and plasticity (Kincses et al., 2004; Fregni et al., 2005).

The precise memory processes that were modulated in our study are elusive. The observed NM did not directly enhance memory encoding per se since the high γ modulation was observed on the poor encoding trials with words that were subsequently forgotten. It could rather enhance memory performance through an associated process. Selective attention, perception and computation of sensorimotor information were all proposed as functions of γ oscillations (Singer, 1993; Tallon-Baudry and Bertrand, 1999; Jensen et al., 2007; Fries, 2009), which are essential to memory performance. If stimulation worked by increasing the level of attention and/or sensory processing of words, it would aid their encoding but not necessarily improve the retention and recall of all of them. In this scenario, the likelihood of successful memory encoding would be increased specifically on the trials with words that were not adequately attended and processed. As a result, more of these words would end up being recalled due to this enhanced attention or perception to the verbal stimuli, which is what we observed on the behavioral level. There would still be words that did not end up being recalled despite the stimulation-induced enhancement of these associated processes. In summary, stimulation would restore processing of these allegedly “;less attended” words, increasing their subsequent recall probability that would lead some, but not all, to transition and add to the number of the recalled words (the good encoding group). Disentangling this challenging relationship between memory and the associated processes requires additional experiments that can track attention and sensory processing through other behavioral or autonomic measures, e.g., the eye movements or pupil dilation.

Another way to identify the cognitive processes modulated by electrical stimulation is to test the existing computational models of memory. One can look for example at the classic primacy and recency effect in remembering lists of stimuli (Murdock, 1962) or the temporal contiguity effect (Sederberg et al., 2010). The former model incorporates serial position of a word on the presented lists with a prior knowledge that the ones in the beginning and in the end of the list tend to be more attended, and thus better recalled, than the middle-list words. The latter is a model of the probability of recall based on temporal proximity of the presented words; words presented next to each other are more likely to be recalled together. In the current paper, we explored these possibilities and did not find compelling evidence for either; however, the current study is limited by a small number of trials to compare. Both of these models may prove useful in future for elucidating the effect of stimulation on memory processing with larger number of subjects.

Finally, physiologic mechanisms of the high γ modulation and how it is linked with the associated behavioral effect remain to be explored. Direct brain stimulation is thought to primarily activate neuronal axons rather than cell bodies (Perlmutter and Mink, 2006), which would provide one explanation for why the electrophysiological effects were observed and not only in the region of stimulation but also in more distant islands of high γ activity, presumably connected with each other. It could also account for the disparity between the frequency of stimulation (50 Hz) and the higher frequencies of the modulated high γ response. Axons of the stimulated white matter tracts may be depolarized and trigger a response of neuronal assemblies oscillating at other frequencies in the distant brain regions they connect. Supporting evidence for the role of axonal stimulation comes from micro-electrode stimulation combined with calcium imaging that shows wide-spread activation of sparsely distributed neurons instead of local depolarization of neurons surrounding the stimulating electrode (Histed et al., 2009).

We observed that the modulation was stronger on electrodes closer to the stimulation site and more active in the tasks. This may possibly reflect a small-world network organization of the brain (Bassett and Bullmore, 2006), which proposes higher number of local and fewer long-distance connections. Therefore, more of the short-range local connections would be depolarized by the electric current and activate more proximal neuronal assemblies, relative to the longer-distance assemblies. In this network view of brain modulation, stimulation would also exert the strongest effect when applied to brain regions, which were critical nodes, i.e., hubs, with many connections to other active nodes in a given network. The lateral TC and the HP, where we observed the strongest positive and negative modulation of high γ activities respectively, are both considered critical hubs for declarative memory networks. Therefore, finding and targeting these critical connection hubs to modulate the whole network instead of a single brain region may be the most efficient strategy for enhancing memory processes (Kim et al., 2016). In our study, stimulation in the lateral TC could work by activating a network hub for verbal declarative memory. These network hubs can potentially be more effectively identified using various measures of connectivity and temporal interactions like spectral coherence or cross-frequency coupling. Future investigations of the brain connectomics data and modeling tools combined with high-density electrophysiological recordings promise to shed light on the mechanisms of electrical modulation for memory and cognitive enhancement.

## Synthesis

Reviewing Editor: Morgan Barense, University of Toronto Decisions are customarily a result of the Reviewing Editor and the peer reviewers coming together and discussing their recommendations until a consensus is reached. When revisions are invited, a fact-based synthesis statement explaining their decision and outlining what is needed to prepare a revision will be listed below. The following reviewer(s) agreed to reveal their identity: Geoffrey Woodman

The reviewers and I were all enthusiastic about the paper's potential for important scientific and clinical impact. However, each reviewer identified some issues (see below), which we all agreed should be addressed in a revision. I look forward to receiving your revised manuscript.

Reviewer 1:

This manuscript reports the findings of experiments in which patients undergoing intracranial electrophysiological observation performed free-recall tasks. The 23 patients received 50Hz stimulation to one brain area (although the text says 22) during the presentation of a couple words during the list. The iEEG/LFPs was also measured and the paper reports that it was the change in high gamma (62-118 Hz) that seems to account for improved memory performance, in the subjects that showed such an improvement.

This is a very interesting dataset. I find the results to be exciting and counterintuitive. The idea that the temporal lobe stimulation was more effective than stimulation of the hippocampus and surrounding bits is particularly surprising and potential important for anatomically based models of memory. I believe that the combination of stimulation and recording is very useful.

The drawback of the paper is that some of the details need to be fleshed out.

1) The paper reports that the data were bandpass filtered from 62-118 Hz to remove line noise. But then it reports findings outside this band, although these are not plotted in the figures. I am not sure how the paper is using bandpass. Usually people mean the frequencies between the low and high cutoff are passed, with the rest filtered. That must not be what the authors mean.

2) Instead of all of the figures only showing 62-118 Hz, please show us the full frequency spectrum in the power plots.

3) Were other frequencies tried that did not have these effects?

4) The small worlds discussion at the end has the reader wondering if cross frequency coupling is increasing, particularly at those distant locations in which high gamma increases. If gamma power is increasing without the theta(or other low)/gamma coupling increasing, this would see to be extremely relevant for the communication-by-coupling hypothesis that guides much of this work looking at time-frequency content.

5) It would be useful to hear a more theoretically grounded proposal for what temporal lobe stimulation is doing. Is it improving the fidelity of the contextual representation at encoding? If so is the temporal contiguity effect stronger? I believe that it would be possible to propose some possibilities in terms of computational models of memory that could be ruled out or supported with analyses of these data.

Reviewer 2:

This paper reports an electrophysiological study of the effect of cortical stimulation on performance of a visual word list learning task. Subjects were epilepsy surgery patients implanted with surface or depth electrodes and cortical stimulation was given through different pairs of electrodes during the encoding phase of the task. Such studies are difficult to achieve due to the scarcity of patients like this and the technical challenges of executing stimulation under these circumstances. At least two sessions were recorded for each stimulation target. Sessions consisted of 25 blocks. During each block subjects viewed and encoded 12 words presented sequentially for 1600 ms each, followed by a distractor task, then free recall of the words in the list. Low intensity cortical stimulation was given in trains that spanned pairs of words (one half of the words in each list with stimulation). Words without stimulation in blocks without stimulation (5) and in blocks with stimulation (20) served as controls. The authors investigated the effect of stimulation on spectral modulations recorded from the rest of the electrodes during encoding of words that were or were not subsequently recalled. The main finding was that high gamma responses to word presentation was modulated by stimulation. This was most evident for trials with poor encoding (no subsequent recall of the word). The effect of stimulation on both behavioral performance and high gamma responses depended on the brain region where stimulation was given. Given the intense general interest in the neurophysiology of memory and the potential of brain stimulation as a therapeutic tool to restore memory function, this paper has the potential to make an important scientific and clinical impact.

Major comments:

One potentially significant weakness of the experimental design is that each of the words remained on the screen for 1600 ms. Spectral power was measured in 50 ms windows over this entire 1600 ms epoch. The subjects could have read and encoded the word stimuli at any time within the 1600 ms, and this could have taken a few hundred milliseconds or could have taken the entire epoch (if the subject was rehearsing the stimuli). If the former, the proportion of time with relevant activation could have been small, diluting any effect from stimulation. In Figure 1, it appears that the activation is concentrated immediately after stimulus onset, and the NM is being measured at this time. However, the subjects could have read and encoded the stimuli at later times in some trials. Also, it looks like Pstim and Pnon could have been drawn from different times. The authors should address this in the Methods.

In the Results, the authors say that stimulation modulated HG power on electrodes showing induced responses to word presentation, which was associated with enhanced memory performance. However, this enhancement was only seen with poor encoding trials (Fig 1c), i.e. with words that were not recalled. In the Discussion it is mentioned that the modulatory effect on 'good' encoding trials was negative (Fig 3c). The authors interpret their results as evidence that stimulation enhanced high gamma, resulting in better memory performance, but this is counterintuitive if the enhancement is only seen with 'poor' encoding trials, i.e. when the word was not subsequently recalled. The effect is also illustrated in Fig 2a and 2b. It may help to describe this differently in the second paragraph of the Results, explaining that poor recall was associated with lower high gamma responses and that stimulation restored these responses to the magnitude seen in good trials, even though the words were not subsequently recalled.

In the Discussion, the authors interpret their findings in light of a previous paper by Ezzyat et al. The authors interpret their results as evidence that brain stimulation is more likely to restore underperforming memory encoding than to enhance good performance, based on the previous study's classification of good and poor encoding states according to induced high gamma. However, their evidence for stimulation enhancing gamma is restricted to trials in which subsequent recall failed, so there is no direct evidence for enhancement of encoding. What results from this experiment could have shown this more directly? It seems that the claim of enhanced performance relies on the recall of words during which no stimulation was given, within the blocks that had stimulation. Yet, the authors do not explicitly acknowledge this or attempt to explain the neurophysiological mechanism that could be responsible for this temporally indirect effect of stimulation.

The Discussion regarding the dependence of stimulation effects on the location of stimulation is very good. However, it would have been very helpful if the authors had reported the results of any cortical stimulation mapping of language that was done in the patients. If it was not done, this should be acknowledged. This data would be useful to better understand the relationship of the stimulation site to cortical language areas. For example, the stimulation sites in 1050 and 1177 are near or in posterior superior and middle temporal gyri, which have been shown in other studies to be important for phonological and lexical representations of words. Were the sites with high gamma enhancement stimulated during language mapping, and if so, what were the results?

Minor comments:

The subject numbers are confusing. It may be better to number them 1-22.

On line 81, it would help to mention which frequencies were used to stimulate in the referenced studies since the gamma range is defined differently by different investigators. Was it the same 50 Hz used by the authors in this study? Later the authors mention that 50 Hz was used in the Suthana and Fried paper, but what about the Kim paper?

Although stimulus intensity was relatively low at 3.0/1.5 mA, this current was delivered in trains lasting 4.6 seconds. The authors should mention whether they observed afterdischarges during or after these trains, either in the stimulating electrodes or the rest of the electrodes. This is an important safety consideration that is more likely to be relevant to replication studies than exceeding safe charge density limits as referenced.

Line 558 should read trials instead of trails.

Is Line 591 supposed to read “and inversely” instead of “in inversely”?

In Methods, the authors say the spectral power values were z-score transformed to normalize across sessions and subjects. What was the rationale for normalizing across subjects? Was this done in lieu of normalizing to a pre-stimulus baseline within subjects?

## Author Response

Dear Editors of eNeuro,

We appreciate excellent comments provided by both reviewers, and addressing them significantly increased the quality of our manuscript. In the following response we provide a detailed response to each concern raised by the reviewers, and how the concerns are addressed in the revised manuscript. Each review comment is re-stated in bold with our response below, including specific changes to the manuscript and figures.

Yours faithfully, Michal Kucewicz

REVIEWER 1

1) The paper reports that the data were bandpass filtered from 62-118 Hz to remove line noise. But then it reports findings outside this band, although these are not plotted in the figures. I am not sure how the paper is using bandpass. Usually people mean the frequencies between the low and high cutoff are passed, with the rest filtered. That must not be what the authors mean.

We appreciate the reviewer pointing out this discrepancy and agree the information was not well phrased in the sixth paragraph of Materials and Methods. The reviewer's understanding is correct â the frequencies between the low and high cut-offs were passed, and this was applied independently for each of the four frequency bands studied. As written in the original manuscript, the reader may get an impression that only the frequencies in the high gamma band were band-pass filtered for the analysis. We have now changed the relevant sentence in this paragraph as follows:

To estimate power in distinct frequency bands (high gamma: 62-118 Hz, low gamma: 30-58 Hz, beta: 14-26 Hz, theta/alpha: 6-14 Hz) signals were band-pass filtered between the corresponding cut-off frequencies (Barlett-Hanning, 1000 order) before spectral decomposition to reduce any possible influence of lower frequencies on the power estimate. The cut-off frequencies for the high gamma band were chosen to minimize contamination of the 60 Hz line noise and its first harmonic at 120 Hz. The decomposed spectral power values in a given frequency band were log and z-score transformed in each frequency bin to account for the power law effect and to normalize across sessions and subjects.

2) Instead of all of the figures only showing 62-118 Hz, please show us the full frequency spectrum in the power plots.

We agree with the reviewer that it is important to show the full spectrum of changes, even if the effects that we report were observed only in the high gamma frequency band. The challenge with showing the full frequency range in the spectrograms is related to the previous point raised by the reviewer about independently band-pass filtering the four frequency bands used in this study before the spectral decomposition. Spectrograms of the raw broad-band signal are dominated by the considerably higher amplitude of the low frequency power, which makes clear presentation of the higher gamma frequencies challenging. The procedure of independent band-pass filtering used in this study before estimating spectral power in the given bands provides a way to show the changes in the gamma frequency band.

We now provide a new Figure 1 (added below for reviewer's convenience) that addresses the reviewer's recommendation to show the entire spectrogram as combined responses in specific bands. The new subplots added to complement Fig. 1c spectrograms, in which power changes in the lower frequency bands (low gamma, beta, theta/alpha) are now plotted independently after band-pass filtering between the corresponding cut-off frequencies. For reviewer's reference, below Figure 1 we also plotted here the full spectrogram with normalized power changes from the same electrode but without the preceding filtering in specific bands. In this new version of Figure 1, all four bands have their power changes presented in the same scale (z-score from -0.5 to 0.5). Thus, the four bands can now be directly compared, despite the amplitude differences related to the one-over-frequency power effect in the original raw signal. We feel that this change is an improvement to data presentation and will help the reader understand the data processing methodology as pointed out in the reviewer's first comment. Please note that a different electrode was chosen as a representative example of the spectral power changes, as specified in the legend. The previous example was chosen to illustrate the largest difference between the STIM and the NON-STIM lists in the âPOORâ MEMORY condition, but was not representative of the entire sample of active electrodes. The new example is more representative with a relatively smaller positive difference in the âPOORâ condition (closer to the median sample value) and a small negative difference in the âGOODâ condition. This example more appropriately reflects the results of the group level analysis in Figure 3c, showing a significant positive change of high gamma band in the former and a non-significant negative change in the latter task condition.

Compared across the four studied bands, the relative power changes reveal a clear general pattern as shown in the Fig. 1c example below. Gamma bands show increased power in response to word presentation, which is of greater magnitude in the high gamma compared to the low gamma band. In contrast, the lower frequencies in the beta and alpha/theta band show an opposite pattern of initial decrease in power when the words are presented, which is followed by slightly increased power. Only the high gamma band showed a consistent effect of stimulation in the âPOORâ condition. There are some differences between the STIM and NON-STIM conditions in the lower bands in this particular example but they are less robust in general and were not significantly different to the other bands as quantified in the Figure 3c analysis. For this reason we decided to focus this manuscript on the high gamma band and suggest not to plot the lower frequency band changes again in Figure 4. In summary, we have now added minor revisions to the second paragraph of Results, according to the changes made in Fig. 1 and legend.

Figure 1. Free recall tasks to study electrophysiological modulation of verbal memory encoding. (a) Diagram of the task design, in which subjects memorized word lists for subsequent recall. Thunderbolt marks the words with stimulation on the âSTIMâ lists. The remaining word trials were used for electrophysiological analysis and are labelled according to the lists type (NON-STIM or STIM) and their encoding based on subsequent recall (âGOODâ or âPOORâ). (b) Example of an 8x8 electrode grid implanted over the lateral temporal cortex highlights in red two adjacent contacts used for brain stimulation (connected red dots) in subject 1050. (c) Spectrogram shows trial-averaged power changes in four frequency bands aligned to the time of word presentation for encoding, as recorded from a representative electrode example from subject 1111. Line plots on the right summarize the mean power change response independently four the four bands (rows) separately for the good and poor encoding trials (columns), in the two conditions of list stimulation, color-coded as in âaâ. Notice the difference in peaks of the response (NM index) caused by stimulation in the poor encoding trials specifically in the high gamma frequency band.

Extra figure of the broadband spectral power changes as in Fig.1c but without independent filtering in specific frequency bands. Notice the lower signal-to-noise ratio, influence of line noise at the 60Hz frequency bin, and less clear boundaries between the frequency bands studied.

3) Were other frequencies tried that did not have these effects?

Yes â systematic analysis of the effect of stimulation on the power changes in the âPOORâ and âGOODâ memory conditions are summarized in Figure 3c. One-way ANOVA revealed significant differences between the four bands in the âPOORâ but not the âGOODâ memory encoding condition as stated in the fourth paragraph of the Results. The profile of these changes in the NM index across the four bands appears in agreement with their respective changes in power as now presented in the Fig. 1c example â low frequency bands show negative effects of stimulation on the power changes and the NM index, in contrast to the higher gamma frequency bands showing a significantly different positive change of the high gamma band in the âPOORâ memory encoding condition (Fig. 1c & 3c). Hence, all subsequent analysis from Figure 3 onwards was focused on this frequency band.

In this study, we have not quantified changes in the lowest frequency bands between 1 and 5 Hz (low theta/delta for several reasons. First of all, high-pass filtering of the electrophysiogical recordings was different across the data collection centers involved in this collaborative project. The cut-offs were set at different values with a selection of filters. Secondly, power changes in the lower frequency bands were in general less robust as compared to the gamma bands (see the new Fig. 1c for an example). This effect was inversely proportional to the frequency, i.e. the lower the frequency, the less consistent the power changes induced in the task. Finally, changes in these low frequency oscillations are more sensitive to modulations in the general arousal states and to epileptiform discharges in the delta range. They may also be more affected by medications applied in this patient population throughout the seizure monitoring procedure. These are, however, important frequency bands to consider in a more controlled future study. Hence, we added the following sentence to the sixth paragraph of the Materials and Methods:

Frequency bands in the low theta and delta ranges between 1 and 5 Hz were not included in this study due to different high-pass filters applied in signal acquisition across the data collection centers.

4) The small worlds discussion at the end has the reader wondering if cross frequency coupling is increasing, particularly at those distant locations in which high gamma increases. If gamma power is increasing without the theta (or other low)/gamma coupling increasing, this would seem to be extremely relevant for the communication-by-coupling hypothesis that guides much of this work looking at time-frequency content.

The reviewer makes an important comment about exploring the effect of stimulation on brain network connectivity, in light of the widespread modulation of the high gamma activity observed in individual electrodes. This is also the focus of our current efforts in the project to better identify potential hubs of brain networks to stimulate. Systematic analysis of connectivity in this patient population is limited by several factors related to the implanted brain regions: limited coverage and number of electrodes typically focused on one specific lobe, various geometrical arrangement of electrodes relative to the cortical layers (subdural surface electrodes may overlie a cortical gyrus or sulcus, depth electrodes are implanted throughout different layers) and heterogenous distribution of epileptiform discharges and other seizure-related activities over the sampled brain areas. These pathological activities may themselves differentially affect low or high frequency spectral activities, e.g. high frequency oscillations associated with lower-frequency waveforms of epileptiform spikes, which are known to cause spurious increases in various measures of cross-frequency coupling. Nonetheless, we have now looked at spectral coherence and cross-frequency coupling in two of the four patients stimulated in the temporal cortex (1050 and 1111), who had enough active electrodes for pair-wise analysis (8 and 22 electrodes, respectively).

Results of the coherence analysis are displayed in Figure 2 below. For each of the two subjects we determined all possible combinations of the bipolar electrode pairs, excluding pairs sharing the same electrode contact (e.g. LTG4-LTG12 & LTG4-LTG5 pair was excluded). In total, 197 pairs of active electrodes were used in subject 1111, and 18 pairs in subject 1050. Coherence was estimated using the same toolbox (Chronux, Bokil et al. 2010) and procedure used in the main manuscript for estimating spectral power â signals from each bipolar electrode of a given pair were first band-pass filtered before the multi-taper spectral analysis of coherence in the specific frequency band (high gamma, low gamma, beta, theta/alpha). Then the resultant coherogram was transformed into z-score in each frequency bin independently to normalize for the power law effect, as in case of the spectrograms in the original manuscript. Mean coherograms were then obtained by averaging coherograms from all pairs, in each frequency band, as presented in Figure 2.

In general, coherograms from subject 1111 show higher signal-to-noise ratio and more consistent patterns than subject 1050 due to a considerably greater number of available pairs. As expected, we observed greater coherence changes in the lower frequency bands, given the proposed role of e.g. theta oscillations in inter-regional synchronization. There were no major changes observed in the gamma bands congruent with more local synchronization mechanisms proposed for these high frequency activities. Comparison between the four task conditions revealed an interesting effect of stimulation in patient 1111, in which reduced low frequency coherence in the beginning and the end of word presentation in the âpoorâ memory condition (blue) is restored on the corresponding stimulated lists (cyan). This effect on coherence is similar to the effect on the induced high gamma power in the main manuscript â stimulation appears to restore the physiological processes selectively in the âPOORâ memory condition. This interesting effect was not observed in subject 1050, which may be due to different localization of the active electrodes (1111 was mainly in the lateral temporal and occipital cortex, whereas 1050 was in the lateral temporal and prefrontal cortex; see Fig. 2 in the main manuscript). The difference may also be subject-specific and at this point we would not feel confident publishing this finding without a replication in more patients. We are very grateful for the reviewer's suggestion to analyze connectivity and make this interesting observation, which will pursue in a larger group of patients stimulated in the temporal cortex.

Figure 2. Stimulation of the lateral temporal cortex has differential effects on coherence across the high and low frequency bands. Upper and lower panels show changes in spectral coherence for subject 1111 and 1050, respectively, in the four color-coded stimulation conditions (columns) and the four studied frequency bands (rows; high gamma, low gamma, beta, alpha/theta).

In addition, we have also tried two different measures of cross-frequency coupling to probe any possible interactions between the low and high frequency activities, as suggested by the reviewer. A Matlab toolbox for quantifying phase-amplitude interactions was used (Onslow, et al. 2011, âQuantifying phase-amplitude coupling in neuronal network oscillationsâ Progress in Biophysics and Molecular Biology 104) to estimate the âModulation Indexâ (Canolty, et al. 2006, âHigh gamma power is phase-locked to theta oscillations in human neocortexâ Science 313) and âCross Frequency Couplingâ (Osipova, et al. 2008 âGamma power is phase-locked to posterior alpha activityâ PLoS One 3) for each bipolar electrode pair. We found no consistent evidence for theta-gamma or any other low-high frequency coupling even within the same electrode pair. There are several possible reasons for this, but arguably the most compelling one is the differences in the task paradigm compared to e.g. the study by Canolty et al., which used the same intracranial electrode recordings in a working memory task. Our paradigm was designed for short-term encoding of verbal information and was not probing the memory maintenance period. The classic model of coupling between the theta and gamma oscillations by Lisman et al. was specifically addressing the problem of buffering working memory information, especially during the maintenance periods. By saying this we are not suggesting that cross-frequency interactions are not engaged in our task at all, but it may be more difficult to study them in the task as currently designed without a clear maintenance phase. The math distractor phase in our task would be expected to reveal more of these interactions as the word lists are held for subsequent free recall. Although analyzing these other phases is beyond the scope of this study, we are planning to investigate them in the near future and our open-access datasets are available to other researchers for further analysis. We have now mentioned these excellent points in the last paragraph of Discussion section in the revised manuscript.

5) It would be useful to hear a more theoretically grounded proposal for what temporal lobe stimulation is doing. Is it improving the fidelity of the contextual representation at encoding? If so is the temporal contiguity effect stronger? I believe that it would be possible to propose some possibilities in terms of computational models of memory that could be ruled out or supported with analyses of these data.

This is another excellent suggestion for more in-depth understanding of our findings. There are two main computational memory models explaining the temporal effects in this particular task. The first one explains the phenomenon of the primacy and recency of stimulus presentation, in which words that are presented towards the beginning and the end of the list have higher probability of being subsequently recalled (Murdock 1962 âThe serial position effect in free recallâ Journal of Experimental Psychology 64). The model assumes that the first and last words are recalled better as subjects tend to pay more attention to these compared to the items presented in the middle of the list. If this effect was not observed in the stimulation condition and the middle-list words were recalled equally well, it would provide a useful insight into the processes that are modulated by the temporal cortex stimulation and ultimately result in memory enhancement. Therefore, we looked into the probability of word recall as a function of its position of presentation in the lists (Figure 3 below, upper panels). For each subject we identified the list position of every recalled word and summarized their counts on the lists with and without stimulation (red and black colors resp. in Fig. 3). The counts were normalized as proportion of the total number of words recalled. In general, all four subjects showed a primacy effect with higher probability of recall for the first few presented words. All but 1176 also showed the recency effect for the last few words. We did not observe any consistent differences between the two stimulation conditions. Subjects 1050 and 1176 did not have enough words recalled resulting in a few instances when there were no words recalled at a particular position in the âblackâ condition of no stimulation (position 6 &8 for subject 1050, and position 4 & 8 for 1176). Therefore, this analysis would benefit from a greater number of trials within each subject (as in 1111 & 1177), and more subjects in general to test the hypothesis on a group level.

The second model of the temporal effects in this task explains why words that are presented right next to each other in time have a higher probability of being recalled together. Multiple studies in rodents have shown that events that occur proximal to each other in time are encoded by overlapping ensembles of neurons (e.g. Malvache et al. 2016, âAwake hippocampal reactivations project onto orthogonal neuronal assembliesâ Science 353). Therefore, recalling one item would be predicted to increase the recall of temporally contiguous item through activation of these common ensembles. Evidence for this temporal contiguity effect in our task was previously reported by the co-authors of this study (Sederberg et al., 2010 âThe temporal contiguity effect predicts episodic memory performanceâ Memory & Cognition 38), showing that the word presented directly before or after a recalled words had the highest probability of being recalled together on a given list.

Figure. 3 Temporal effects of word presentation on memory recall in the stimulated (red) and control (black) list conditions are summarized for the four subjects with lateral temporal cortex stimulation. Upper panels show the primacy and recency effects of word presentation, and the lower panel shows the proportion of recalled words presented directly next to each other on the word lists.

Hence, we checked whether the proportion of the recalled words separated by one list position (either before or after) would change as a function of the stimulation (Fig. 3 above, lower panel). If the stimulation was working by enhancing contextual encoding of words, one would expect to see an increase in the proportion of words presented directly next to each other on the lists. Even though we observed an increased proportion on the stimulated lists in three out of the four subjects, the magnitude of this effect was modest except for subject 1177. Subject 1176 showed an opposite pattern of a decreased proportion on the stimulated lists. As in the case of the first model, this exciting analysis requires a greater number of subjects to assess the effect of stimulation. The study by Sederberg et al. used âNâ numbers in the order of 100s of subjects to characterize the temporal contiguity effect. The advantage of having this model does not require us to use as high âNâ numbers but a group of at least 10 subjects will be needed to address these important questions in the future studies.

We have now added an additional paragraph to introduce the suggestions made by the reviewer as the new paragraph no. 7 of Discussion with two references included (Murdock and Sedeberg) as discussed above. The paragraph now states:

Another way to identify the cognitive processes modulated by electrical stimulation is to test the existing computational models of memory. One can look for example at the classic primacy and recency effect in remembering lists of stimuli (Murdock et al., 1968), or the temporal contiguity effect (Sederberg et al. 2010). The former model incorporates serial position of a word on the presented lists with a prior knowledge that the ones in the beginning and in the end of the list tend to be more attended, and thus better recalled, than the middle-list words. The latter is a model of the probability of recall based on temporal proximity of the presented words â words presented next to each other are more likely to be recalled together. In the current paper we explored these possibilities and did not find compelling evidence for either, however, the current study is limited by a small number of trials to compare. Both of these models may prove useful in future for elucidating the effect of stimulation on memory processing with larger number of subjects.â

REVIEWER 2

1) One potentially significant weakness of the experimental design is that each of the words remained on the screen for 1600 ms. Spectral power was measured in 50 ms windows over this entire 1600 ms epoch. The subjects could have read and encoded the word stimuli at any time within the 1600 ms, and this could have taken a few hundred milliseconds or could have taken the entire epoch (if the subject was rehearsing the stimuli). If the former, the proportion of time with relevant activation could have been small, diluting any effect from stimulation. In Figure 1, it appears that the activation is concentrated immediately after stimulus onset, and the NM is being measured at this time. However, the subjects could have read and encoded the stimuli at later times in some trials. Also, it looks like Pstim and Pnon could have been drawn from different times. The authors should address this in the Methods.

The reviewer is absolutely right about the challenge of determining the time when memory is encoded in the task. Not only we would expect variable timing of the encoding processes between different subjects (each individual is predicted to develop specific strategies for encoding and rehearsal of the encoded information) but also within any one subject we would expect different latencies and responses between words and across lists. In addition, a subject may develop preferences for particular categories of words e.g. animals, and process them in a faster or different way than others. This variability is actually accordingly reflected in the proxy of brain activation that we decided to use in this task â peak of the power response. In the example from Fig. 1c, it is evident that the average high gamma response to word presentations is not very sharp around a specific latency but rather âsmearedâ around the peak maximum with a longer tail towards greater latencies. This average profile is a composite of all resposes to individual words, which âon averageâ peak around 500ms Â± 200ms. It seems likely that these variable responses may reflect the variable timing of memory encoding in a specific brain structures.

In a different set of ârecord-onlyâ experiments without any stimulation we looked into these latency patterns in more detail taking advantage of a large dataset of 184 patients. Figure 4 below summarizes the main results of this analysis, which used approx. 1000 active electrodes identified in 16 different Brodmann areas, color-coded according to the cortical lobe. One can see a clear pattern of progressively longer latencies of the high gamma response to word presentation starting from the early sensory areas of the occipital cortex, through to higher order associational areas in the frontal lobe. The peak response is not only gradually delayed in time but also less sharp and and thus of lower amplitude in different regions of the hypothetical visual processing streams (Goodale & Milner 1992 âSeparate visual pathways for perception and actionâ Trends in Neuroscience 15). The cyan and purple average responses recorded in the frontal lobe show greater spread in time as perhaps might be expected of the processes engaged in these higher-order areas compared to more stereotypical responses in the more sensory regions of the occipital cortex.

Figure 4. Comparison of the high gamma responses to word encoding in the free recall tasks across 16 distinct Brodmann areas. Left side panel shows mean power responses from at least 25 different active electrodes localized in a specific Brodmann area and color-coded according the cortical lobe. Right side panel summarizes the latencies of the peak maximum value of each curve from the left-side plots. This data was presented on the last meeting of the Society for Neuroscience is prepared for publication.

Therefore, peak of the average power response admittedly does not provide a very accurate estimate of memory encoding but rather a more coarse measure, which accomodates for the different timings of the response in particular brain regions. The right side panel of Fig. 4 shows that latencies of these peaks in the studied Brodamann areas spanned almost the entire epoch of word presentation from 200ms through to over 1500ms after onset. It worth noting, however, that most responses happened in the first 500ms after onset speaking for decreasing the length of word presentation in a future design. All things considered, this is why we decided to use the entire period of memory encoding to find the peak values and based on them determine the NM index, as described in the Methods section (see revised below), without any apropri assumptions about the exact timing of memory encoding.

Based on the unpublished results presented in Fig. 4 above, we are now preparing a manuscript focused on the spatiotemporal patterns of the high gamma responses and their relationship with memory encoding. This important point has now been explained in the seventh paragraph of Methods section:

Exact time of memory encoding during the stimulus presentation is difficult to determine and can vary between subjects. We used the maximum peak value of the average power estimates as proxy for the brain response related to the memory encoding. This maximum value of the average power estimate was defined as peak power, and the difference between peak power values from the stimulated (Pstim) and non-stimulated (Pnon) list condition was defined as the âNeuromodulation (NM) indexâ

And in the second paragraph of Results section:

The modulatory effect of stimulation was quantified as a difference between peaks of the power response in the âSTIMâ minus the âNON-STIMâ condition, which we called the neuromodulation (NM) index (Fig. 1c). The peak response was thus used as proxy for brain activity related to memory encoding.

â

2) In the Results, the authors say that stimulation modulated HG power on electrodes showing induced responses to word presentation, which was associated with enhanced memory performance. However, this enhancement was only seen with poor encoding trials (Fig 1c), i.e. with words that were not recalled. In the Discussion it is mentioned that the modulatory effect on 'good' encoding trials was negative (Fig 3c). The authors interpret their results as evidence that stimulation enhanced high gamma, resulting in better memory performance, but this is counterintuitive if the enhancement is only seen with 'poor' encoding trials, i.e. when the word was not subsequently recalled. The effect is also illustrated in Fig 2a and 2b. It may help to describe this differently in the second paragraph of the Results, explaining that poor recall was associated with lower high gamma responses and that stimulation restored these responses to the magnitude seen in good trials, even though the words were not subsequently recalled.

The effect of stimulation in trials with the words that were subsequently recalled (âgoodâ encoding) and forgotten (âpoorâ encoding) was indeed not clearly explained in the second paragraph of Results. We are grateful that the reviewer spotted this critical point for explaining our findings. We have now revised that section by adding the following statement:

The high gamma response on trials with words that are subsequently not recalled (âpoorâ encoding) is known to be decreased relative to trials with the subsequently recalled words (âgoodâ encoding), as previously described (Kahana 2006, Sederberg et al., 2007). Stimulation on the âpoorâ encoding trials increased this high gamma response on the âpoorâ encoding trials and restored it to the magnitude observed on the âgoodâ encoding trials with words that were subsequently recalled (Fig. 1c). Thus, the subsequently forgotten words from the stimulated âSTIMâ lists had increased high gamma response relative to the words from the âNON-STIMâ lists that were not stimulated.

â¦and further clarified this point in the following third paragraph of the Results, describing Figure 2:

Figure 2 presents three exemplar cases of stimulation from subdural surface grid electrodes in the temporal cortex, which modulated the peak power responses. The top case depicts a single discrete area of the peak activation. The magnitude of this discrete high gamma response is greater on the âgoodâ than the âpoorâ encoding trials in the NON-STIM control condition â this disparity between the remembered and the forgotten word trials is not present in the STIM condition with similar peaks on the two trial types (Fig. 2a). Stimulation therefore increased the high gamma response on the poor encoding trials to the levels seen during âgoodâ encoding, selectively in the area of the induced task activity.

â

3) In the Discussion, the authors interpret their findings in light of a previous paper by Ezzyat et al. The authors interpret their results as evidence that brain stimulation is more likely to restore underperforming memory encoding than to enhance good performance, based on the previous study's classification of good and poor encoding states according to induced high gamma. However, their evidence for stimulation enhancing gamma is restricted to trials in which subsequent recall failed, so there is no direct evidence for enhancement of encoding. What results from this experiment could have shown this more directly? It seems that the claim of enhanced performance relies on the recall of words during which no stimulation was given, within the blocks that had stimulation. Yet, the authors do not explicitly acknowledge this or attempt to explain the neurophysiological mechanism that could be responsible for this temporally indirect effect of stimulation.

This is another important point, which relates to the previous one and requires further clarification in the manuscript. The reviewer is right about lack of direct evidence for an enhancement of memory encoding that would provide an explanation for the observed behavioral enhancement of memory performance in these free recall tasks. This is the reason why we speculated in the sixth paragraph of Discussion that the positive effect of stimulation in the lateral temporal cortex was probably not achieved by modulating memory encoding directly, but rather an associated process related to attention or perception of the stimuli:

The precise memory processes that were modulated in our study are elusive. The observed neuromodulation may not enhance memory encoding per se, but rather an associated process. Selective attention, perception and computation of sensorimotor information were all proposed as functions of gamma oscillations (Singer, 1993; Tallon-Baudry and Bertrand, 1999; Jensen et al., 2007; Fries, 2009) , which are essential to memory performance.

We have now revised this paragraph to more explicitly state the reviewer's point:

The precise memory processes that were modulated in our study are elusive. The observed neuromodulation did not directly enhance memory encoding per se since the high gamma modulation was observed on the âpoorâ encoding trials with words that were subsequently forgotten. It could rather enhance memory performance through an associated process. Selective attention, perception and computation of sensorimotor information were all proposed as functions of gamma oscillations (Singer, 1993; Tallon-Baudry and Bertrand, 1999; Jensen et al., 2007; Fries, 2009), which are essential to memory performance.

And then we further revised the remaining part of this paragraph and removed the reference to Ezzyat et al. for the sake of clarity:

If stimulation worked by increasing the level of attention and/or sensory processing of words, it would aid their encoding but not necessarily improve the retention and recall of all of them. In this scenario, the likelihood of successful memory encoding would be increased specifically on the trials with words that were not adequately attended and processed. As a result, more of these words would end up being recalled due to this enhanced attention or perception to the verbal stimuli, which is what we observed on the behavioral level. There would still be words that did not end up being recalled despite the stimulation-induced enhancement of these associated processes. In summary, stimulation would restore processing of these allegedly âless attendedâ words, increasing their subsequent recall probability that would lead some, but not all, to transition and add to the number of the recalled words (the âgoodâ encoding group). Disentangling this challenging relationship between memory and the associated processes requires further experiments that can track attention and sensory processing through other behavioral or autonomic measures, e.g. the eye movements or pupil dilation.

We also revised the second paragraph of the Discussion, which compares our results to the previous study by Ezzyat et al. 2017. We propose to keep this useful reference here but conclude the paragraph with the point made by the reviewer instead of the previously used statement about ârestoring underperforming processes of memoryâ (see below). We agree with the reviewer that our reported effect requires further investigation, which is not clear at this point. Since we are in agreement with the reviewer on this point, any edits to this text that would help clarify this issue even more are most welcome. The corresponding text now reads:

In a recent study of electrical stimulation applied during word encoding, the induced high gamma activity was used to classify brain states into âgoodâ and âpoorâ encoding states and predict that stimulating in the âgoodâ state decreased the probability of recall and vice versa increased probability of recall when stimulating in the âpoorâ encoding state (Ezzyat et al., 2017). This interesting finding of âgoodâ and âpoorâ encoding state-dependency is consistent with our observation of a positive stimulation-induced NM index during the âpoorâ encoding trials and a negative index during the âgoodâ trials. Still, the positive effect of stimulation on the high gamma activity was restricted to trials with words that were ultimately forgotten, making it challenging to explain the overall enhancement observed in the increased number of recalled words.

â

4) The Discussion regarding the dependence of stimulation effects on the location of stimulation is very good. However, it would have been very helpful if the authors had reported the results of any cortical stimulation mapping of language that was done in the patients. If it was not done, this should be acknowledged. This data would be useful to better understand the relationship of the stimulation site to cortical language areas. For example, the stimulation sites in 1050 and 1177 are near or in posterior superior and middle temporal gyri, which have been shown in other studies to be important for phonological and lexical representations of words. Were the sites with high gamma enhancement stimulated during language mapping, and if so, what were the results?

We agree with the reviewer that the information about mapping the language functions in specific patients is essential to this study. In addition, it would also be useful for the reader to know about any deficits in verbal memory functions that could also play a role in the effect of stimulation. The original Table 1 included only information about seizure the onset zone localization and lesions (structural MRI, brain pathology). Table 1 has now been updated with new columns specifying: the language laterality and the method used to establish it (where available), overlap of the target stimulating electrodes with the electrodes identified during cortical mapping of the language areas (where available), and neuropsychological assessment of verbal memory performance, with the verbal IQ score where available. Table 1 is provided below for reviewer's convenience:

Subject No. Age Gender Handedness SOZ MRI Brain pathol. Language laterality

(method) Stim.

mapping overlap vIQ Verbal

memory

deficits

1001 48 F R right TC Normal Gliosis L (fMRI) - 81 None

1006 20 F R right FC MCD Gliosis L (fMRI) - 91 None

1016 31 F R left FC Normal Gliosis - None 71 None

1018 47 M L left FC,

left FPC Normal - L (fMRI) - 85 None

1020 48 F L right TC,

right FC Abnormal Gliosis L (fMRI) - 98 Mild

1022 24 M R Atrophy

Gliosis/

Encephalomalacia - L (fMRI) - 81 None

1024 36 F R right OPC Normal Gliosis L (unknown) - 100 None

1026 24 F R left aTC

left OC MTS, Gliosis - Bilateral (Wada) - 112 None

1027 48 M R right TC right IC

right/left FC

Abnormal - L (fMRI) - 93 None

1028 27 F R right MTL Abnormal CD, Gliosis L (Wada) - 103 None

1029 33 F R left FC Abnormal - - - 108 Mild

1030 23 M L left MTL Normal Gliosis L (fMRI) - 106 None

1031 24 M R right FC right TC Abnormal - L (aphasia) - 110 Moderate

1033 31 F R right TC Atrophy - L (Wada) - 85 None

1036 49 M L left aTC,

left MTL MTS HS Bilateral (Wada) - 93 Moderate

1042 27 F L right TC MCD - R (fMRI) None 114 None

1050 20 M R left PC Neoplasm DNET bilateral (Wada) None 95 Mild

1060 36 F R right TC Normal Gliosis L (Wada) - 95 Mild

1069 26 M R left FC MCD - L (Wada) - - Mild

1111 20 M R left TC left OPC

left OC Gliosis Gliosis L (fMRI) - 108 None

1176 41 F R right MTL right IC MTS - L (Wada) - 85 Moderate

1177 23 F R left TC TS - L (aphasia) None 87 Moderate

Table 1. Clinical profile of the study participants. Patient demographic data is presented together with clinical observations from structural magnetic resonance imaging (MRI), clinically identified seizure onset zones (SOZ), pathology for those subjects who underwent resective surgery, hemispheric laterality of language functions together with the method of determination (âaphasiaâ means that the determination was done based on an identified lesion/pathology in a specific hemisphere), overlap of the stimulating electrodes with the language areas for patients who have undergone cortical stimulation mapping (â-â means that the stimulation mapping was not performed or the report was not available), verbal IQ (vIQ), and the clinical qualitative description of verbal memory deficits as concluded in the neuropsychological assessment. Abbreviations: FC â frontal cortex, TC â temporal cortex, PC â parietal cortex, OC â occipital cortex, IC â insular cortex, aTC â anterior temporal cortex, MTL â mesial temporal lobe, TPC â temporo-parietal cortex, FPC â fronto-parietal cortex, OPC â occipito-parietal cortex, CD â cortical dysplasia, HS â hippocampal sclerosis, MCD â malformation of cortical development, MTS â mesial temporal sclerosis, PMG â polymicrogyria, DNET â dysembryoplastic neuroepithelial tumor.

Cortical mapping data was available only in four patients due to missing clinical reports or no cortical stimulation mapping performed for the remaining patients. Fortunately, data for the two patients of interest pointed out by the reviewer (1050 & 1177) was available and showed no overlap of the mapped language areas with the target electrodes chosen for the task stimulation. Similarly, we found no evidence for overlap of language areas with the stimulating electrodes in the other two patients available.

With regard to assessing verbal memory performance, verbal IQ was the only quantitative measure that available for almost all patients across the clinical centers engaged in this study. The neuropsychological tests employed differed not only between but also within the clinical centers. Each patient performed a specific set of tasks as part of their individualized neuropsychological assessment. Hence, it was very challenging to identify a common test or measure that could be used across all patients and clinical sites. We therefore had to go through all reports of neuropsychological assessments and summarize conclusions of a given clinician about verbal memory performance. Based on these conclusions, we created the last column of Table 1 with a qualitative grade of verbal memory deficits. Four grades were established: severe, moderate, mild, or none. The last grade was given to the patients who were assessed as average or above average in their verbal memory performance. Using this qualitative measure, all patients were assessed with a neuropsychological evaluation probing verbal memory. In total, 5 patients presented mild deficits, and another 4 were moderate. The remaining 13 patients had no deficits identified. It was interesting that the verbal IQ score does not always correlate with the qualitative assessment of verbal memory performance, which might be explained by the fact that verbal IQ measure more general language performance including fluency, reading and comprehension that are distinct from the ability to memorize verbally presented stimuli.

We are grateful to the reviewer for suggesting to look further into the clinical assessment of the language functions. This now provides a fuller picture to interpret the results of stimulation. The main manuscript text was accordingly updated to make note of these additions in the fifth paragraph of the Materials and Methods section, and in the end of the third paragraph of the Results.

Minor comments:

The subject numbers are confusing. It may be better to number them 1-22.

This study is part of a bigger project with open-access data. For the sake of reproducibility of these results we would like to keep the subject identifier numbers. Nonetheless, we agree that the letter system used with the identifier number is confusing and redundant. We have therefore removed the letters from the identifier numbers in the Tables, figure legends, and the main manuscript text.

On line 81, it would help to mention which frequencies were used to stimulate in the referenced studies since the gamma range is defined differently by different investigators. Was it the same 50 Hz used by the authors in this study? Later the authors mention that 50 Hz was used in the Suthana and Fried paper, but what about the Kim paper?

Since Kim et al. is a review paper summarizing many other we specified a range of frequencies used in all these studies and clarified in the manuscript that this is a review paper. The relevant statement now reads:

âHowever, the reports of a positive effect on memory performance in humans were all stimulating at frequencies in the gamma range (40/50/200 Hz; for review see Kim et al., 2016), suggesting that the applied current presumably modulated similar frequencies of neuronal oscillations.â

Although stimulus intensity was relatively low at 3.0/1.5 mA, this current was delivered in trains lasting 4.6 seconds. The authors should mention whether they observed afterdischarges during or after these trains, either in the stimulating electrodes or the rest of the electrodes. This is an important safety consideration that is more likely to be relevant to replication studies than exceeding safe charge density limits as referenced.

We completely agree with the safety concerns for the future studies and have now added a new statement in the fifth paragraph of Materials and Methods:

âSafe amplitude for stimulation was determined at the start of each session using a mapping procedure in which stimulation was applied at 0.5 mA while a neurologist monitored for after-discharges. This procedure was repeated, incrementing the amplitude in steps of 0.5 mA, up to a maximum of 1.5 mA for depth contacts and 3.5 mA for cortical surface contacts. These maximum amplitudes were chosen to be below the after-discharge threshold and below accepted safety limits for charge density (McCreery et al., 1990).â

Line 558 should read trials instead of trails.

Is Line 591 supposed to read “and inversely” instead of “in inversely”?

We corrected both. Thank you for spotting these typing errors.

In Methods, the authors say the spectral power values were z-score transformed to normalize across sessions and subjects. What was the rationale for normalizing across subjects? Was this done in lieu of normalizing to a pre-stimulus baseline within subjects?

The sentence pointed out by the reviewer has now been rephrased to:

The decomposed spectral power values in a given frequency band were log and z-score transformed in each frequency bin to account for the power law effect and obtain values that can be compared in the same scale across sessions and subjects.

This normalization method uses the mean of the whole signal instead of the pre-stimulus baseline. It effectively quantifies the change from the mean power estimate in the z-score units of standard deviations. This was especially important for the Figure 3c,d and the Fugure 5c,d analyses, which pooled the NM index values from different electrodes within the same subject (Figure 3c,d) and the overall mean NM index values from all electrodes (Figure 5c) and subjects (Figure 5d). It is expected that different amplitudes of the signals recorded from electrodes with a range of impedance values and other biophysical factors, can have an impact of the magnitude of NM index. Normalizing to baseline would also be a good method but in our case we only had 200ms of the pre-stimulus baseline (to avoid artifacts from the preceding stimulated words), which was in most cases already had some preparatory activity (for instance see the decreased high gamma power in the Figure 1c example).
